# Structural connectivity of grandiose versus vulnerable narcissism as models of social dominance and subordination

**DOI:** 10.1038/s41598-023-41098-1

**Published:** 2023-09-26

**Authors:** Lisa Schmidt, Julia-Katharina Pfarr, Tina Meller, Ulrika Evermann, Igor Nenadić

**Affiliations:** 1 Cognitive Neuropsychiatry Lab, Department of Psychiatry and Psychotherapy, Philipps Universität Marburg, Marburg, Germany; 2 Marburg University Hospital – UKGM, Marburg, Germany; 3 Center for Mind, Brain and Behavior (CMBB), University of Marburg and Justus Liebig University, Giessen, Germany

**Keywords:** Neuroscience, Psychology, Medical research

## Abstract

Social dominance and subordination have been linked to fronto-limbic and fronto-thalamic networks and are related to phenotypes such as grandiose vs. vulnerable narcissistic traits. The latter have been linked to clinical features such as empathy and emotional regulation. In this study we tested the hypotheses that narcissistic traits are associated with white matter integrity in fasciculus uncinate, cingulum, and anterior thalamic radiation (ATR). We applied the Pathological Narcissism Inventory (PNI) to assess narcissistic traits in a sample of 267 psychiatrically healthy individuals. We used 3 T MRI to acquire Diffusion Tensor Imaging data for analysis with TBSS in FSL applying TFCE to test for correlations of fractional anisotropy (FA) and PNI scales. We detected a significant positive correlation of PNI total and FA in the right posterior cingulum. PNI Vulnerability was significantly correlated with FA in the left anterior and right posterior cingulum. We did not find overall correlations with PNI Grandiosity, but additional analyses showed significant effects with FA of ATR. Our results strengthen network models for narcissism underlying both personality variation and pathology. Especially associations of narcissistic vulnerability within fronto-limbic tracts suggest overlaps within neural correlates of related phenotypes like neuroticism, social subordination, and negative emotionality.

## Introduction

Social dominance hierarchies are a ubiquitous phenomenon across most species^1^. Recognition, representation, or regulation of social status are essential cognitive processes underlying these hierarchies. In animal models, these cognitive functions are represented in prefrontal brain areas^2^ especially in the medial prefrontal cortex (mPFC^2,3^; and its dorsomedial-thalamic connections^4^. In humans, neural correlates of social dominance are assumed to be represented in fronto-limbic areas, including medial, ventromedial, dorsolateral and cingulate cortices, amygdala, and hippocampus^5–8^. In turn, social subordination is associated with fronto-striatal systems including ventromedial, ventrolateral and insula cortices, and striatum^6,8^.

These behaviors and cognitions are closely related to clinical phenotypes such as narcissism with features such as entitlement, exploitative behavior, inflated self -importance, authoritarianism, self-sufficiency, striving for power^9,10^ and self-aggrandizement^11^.

This phenotype is a diametric construct including grandiose and vulnerable aspects. Grandiose narcissism reflects a pervasive pattern of grandiosity, need for admiration, and unwillingness to empathy, thus its features resembling social dominance. Furthermore, self-importance is highlighted with preoccupied fantasies of unlimited success, power, brilliance, and beauty^12,13^. Vulnerable narcissism which is associated with social subordination orientation is expressed by low and fragile self-esteem, social avoidance, axis I and II psychiatric disorders and general emotional vulnerability^14–16^. Higher prevalence of unpleasant emotional states like anger, aggression, and shame are outlined^16,17^. Entitlement, one’s belief about deserving special benefit and attention is a common feature which is present in both narcissistic facets^18,19^.

Narcissism can be assessed with self-evaluative measurement tools like the Pathological Narcissism Inventory (PNI)^16^ which includes grandiose and vulnerable aspects of narcissism within seven subscales (contingent-self-esteem, devaluing, hiding the self, exploitation, entitlement rage, self-sacrificing-self-enhancement, and grandiose fantasy). The Narcissistic Personality Inventory (NPI)^20,21^ is another questionnaire to measure narcissistic traits but is restricted to grandiose features alone^20^.

So far, only few neuroimaging studies exist on narcissism. However, differences in prefrontal networks^22–25^ were consistently identified, overlapping with brain structures involved in constructs like social functioning, empathy, and emotional regulation^26,27^. Moreover associations with functional activation in cingulate, insular^28^ and premotor cortex^29^, as well as structural correlates in postcentral gyrus^25^ were described. It should be noted that narcissism was differently assessed across studies using PNI^22,25^, NPI^24,28^, Narcissism Inventory (NI)^29^ or clinical judgement^23,30^.

Reduction of white matter integrity in fronto- thalamic pathways were identified in patients with NaPD (Narcissistic personality disorder^22,30^; and are assumed to be relevant in social expectation, supposed to be biased in narcissistic individuals^16^. Moreover, differences in fronto-limbic fiber tracts were demonstrated in NaPD patients, assumed to reflect the deficit in emotion regulation^22,30^. Comparable results were found in other cluster B personality disorders^31,32^. In addition, even in healthy individuals, negative associations of structural connectivity and grandiose narcissistic traits in parts of cortico-striatal-thalamo-cortical (CSTC) networks were found^33^. This is supposed to explain deficits with intrinsically generating positive self-views, which narcissists experience^33,34^.

In this study, we used the PNI to test whether grandiose vs. vulnerable narcissistic traits are associated with white matter integrity in three selected tracts in fronto-limbic and fronto-thalamic networks in a non-clinical sample. We chose the PNI due to its coverage of both grandiose and vulnerable features. We focused on ATR on account of the results of a previously published pilot study of NaPD^30^ and overlaps of this tract with fronto- striatal connections which are associated with sub-clinical grandiose narcissism^33^. We selected UF and cingulum to test for an association with especially vulnerable traits based on neural corelates with related phenotypes like neuroticism^35^ and negative emotionality^36^.

## Methods

### Subjects

We analysed a sample of 296 healthy subjects (107 male, 189 females, mean age = 23.65 years; SD = 3.58) meeting the inclusion criteria, i.e.: age 18–45, native German speaker, central European origin, and normal or corrected-to-normal vision. Exclusion criteria were traumatic brain injury, neurological or CNS (central nervous system) related or psychiatric disorders, psychotropic medication, BMI < 18 or > 35, MR contraindications, and physical disorders incompatible to MR scanning. Furthermore, an estimate of general intellectual ability was obtained using the German Mehrfach-Wortschatz-Intelligenztest B (MWT-B)^37^ to exclude subjects with general intellectual impairment or learning disability (IQ < 80). We used the DSM-IV screening for axis I disorders (SCID; German version)^38,39^ to assure the absence of current or previous psychiatric disorder, psychotherapeutic treatment, substance abuse or dependence. Psychometric and MRI data were obtained within two weeks. Descriptive statistics are illustrated in Table 1. The study was approved by the local Ethics Committee of the school of medicine, Philipps-University Marburg, and conducted according to the current version of the Declaration of Helsinki^40^. All participants gave written informed consent before inclusion in the study and were financially compensated afterwards.

**Table 1:** Descriptive statistics of PNI-scales.

Variable	N	Range	M (SD)	Skewness	Kurtosis	Cronbach’s alpha
Age	267	18–37	23.67 (3.45)	1.00	1.29	
Female	171 (64%)					
Male	96 (36%)					
**PNI Total**	**267**	**1.17–4.17**	**2.57 (0.65)**	**0.14**	** − 0.44**	**0.95**
**Grandiosity**	**267**	**1.21–5.06**	**2.89 (0.73)**	**0.05**	** − 0.12**	**0.88**
**Vulnerable**	**267**	**1.00–4.60**	**2.39 (0.71)**	**0.36**	** − 0.35**	**0.94**
CSE	267	1.00–5.00	2.51 (0.86)	0.41	− 0.40	0.89
EXP	267	1.00–5.71	2.57 (0.86)	0.55	− 0.09	0.85
DEV	267	1.00–4.86	2.06 (0.80)	0.70	0.13	0.82
SSSE	267	1.00–5.50	3.28 (0.92)	− 0.16	− 0.37	0.82
GRAND	267	1.00–5.43	2.75 (0.97)	0.34	− 0.36	0.84
ENTR	267	1.00–4.63	2.35 (0.84)	0.51	− 0.38	0.86
HIDE	267	1.00–5.43	2.64 (0.91)	0.51	− 0.17	0.82

CSE: Contingent-Self Esteem, EXP: Exploitative, DEV: Devaluing, SSSE: Self-sacrificing self-enhancement, GRAND: Grandiose Fantasy, ENTR: Entitlement Rage, HIDE: Hiding the Self.

### Assessing narcissism with pathological narcissism inventory (PNI)

The Pathological Narcissism Inventory (PNI)^16^ served as an assessment tool for grandiose and vulnerable narcissistic features. We used an online version of the German version validated by^13^. The PNI is a 52 item self-report measurement with six-point Likert-Scale (0 = “not at all like me” to 5 “very much like me”). All items can be assigned to two main factors (*Grandiose* and *Vulnerable*) and further allocated to seven sub factors. Traditionally, grandiose narcissism is operationalized by *Entitlement rage* (ENTR; 8 items), *Exploitative* (EXP; 7 items), *Grandiose fantasy* (GRAND; 7 items), and *Self*-*sacrificing*-*self*-*enhancement* (SSSE; 6 items) subscales. These variables describe manipulative, egoistic and megalomaniac behavior and self-boosting strategies^13^. In contrast, *Hiding the self* (HIDE; 7 items), *Devaluing* (DEV; 7 items), and *Contingent*-*self*-*esteem* (CSE; 12 items) scales illustrate vulnerable aspects like fluctuating self-esteem, high dependence on others, and disguising of own needs^13^. The German Version included two new items to expand the grandiose subscale *Exploitative*^13^ because it only consisted of five items in the original version^16^. These items were adopted from the Narcissistic Personality Inventory (NPI)^20,41^ agreed with the higher order two factor model of narcissism from^16^ but plead for another seven-factor solution with *Entitlement rage* as a part of the vulnerable pole because of better model fit.

We examined reliability for all scales (n = 10: *total score*, *Grandiose*, *Vulnerable*, *CSE, DEV, EXP, ENTR, GRAND, SSSE, and HIDE).* PNI *total score* reliability (see Table 1) can be compared to the original English (n = 2801 α = 0.95;^16^) and German sample (n = 1837, α = 0.94;^13^). Reliability for the two main scales *Grandiose* and *Vulnerable was not reported by former studies* however Cronbach’s alpha (α) for the other seven subscales was conducted.

**Figure 1:**
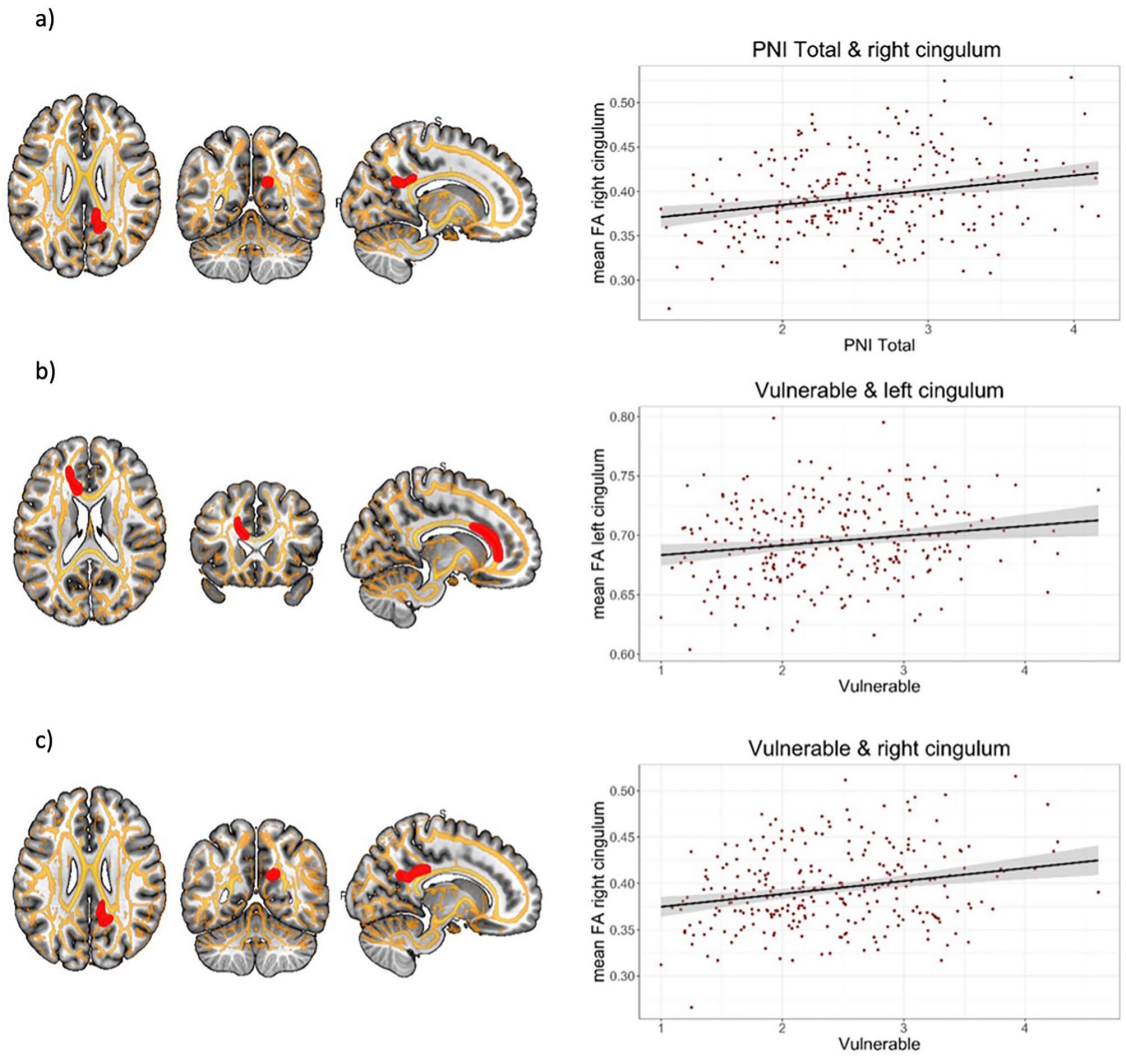
Association of PNI scales and FA (two-dimensional). PNI Total and Vulnerable scale and association with FA (all FWE peak level corrected) and scatter plots (made with R Studio (version 2022.2.3.492, https://www.rstudio.com/); yellow: tract mask; red: significant clusters; cluster edges are displayed enlarged; depiction made with MRICroGl (version 12.3.1, https://www.nitrc.org/projects/mricrogl); (**a**) PNI Total & right posterior cingulum; (**b**) PNI Vulnerable & left anterior cingulum; (**c**) PNI Vulnerable & right posterior cingulum.

We computed scale reliability, Cronbach’s alpha, for PNI *total score* (α = 95), *Vulnerable* (α = 0.94) and *Grandiose* (α = 0.88). Further, we analyzed the seven sub-scales which show high reliabilities, too (CSE α = 0.89, ENTR α = 0.86; EXP α = 0.85; GRAND α = 0.84; DEV α = 0.82; SSSE α = 82; HIDE α = 0.82). Moreover, an inter-scale correlation heatmap is represented in supplemental material (Fig. 1). We did correlations analyses (Table 4) with PNI scales and demographic variables age, sex, education, and occupation. Age was negatively correlated with PNI *total* (r = − 14, *p* = 0.02), *Grandiosity* (r =−0.17, *p* = 01), SSSE (r = − 0.20, *p* < 0.001), GRAND (r = −0.13, *p* = 0.04), and ENTR (r =  − 0. 13, *p* = 0.03). PNI Grandiosity (r = 0.13, *p* = 0.03) and EXP (r = 0.29, *p* < 0.001) were correlated with being male and CSE (r = − 0.17, *p* = 0.01) was correlated with being female. We did not find any significant associations with education. Occupation was positively correlated with PNI EXP (r = 0.12, *p* = 0.04).

### MRI data acquisition

We used a 3 Tesla MRI scanner with 12 channel head matrix Rx coil (Siemens Magnetom, Tim Trio, Erlangen, Germany) to obtain MRI data. An isotropic diffusion weighted EPI 2D sequence with mode MDDW (repetition time = 7300 ms, echo time = 90 ms, slice amount = 56, slice thickness = 3 mm, isotropic voxel resolution = 2.5 × 2.5 × 2.5 mm^3^) was applied for DTI images. 2 × 30 diffusion weighted images along 30 non-parallel directions (b = 1000 s/mm^2^) as well as four non-diffusion-weighted images (b = 0 s/mm^2^) were acquired for every participant. Afterwards, all images were visually controlled for major artifacts and 29 subjects had to be eliminated from DTI analyses because of motion, leaving a DTI sample of n = 267 (171 male, 96 female, mean age 23.67 years, SD 3.45).

### MRI Data pre-processing

We used an established methodological pipeline for MR acquisition and data pre-processing from our lab (Cognitive Neuropsychiatry Lab;^42^). A Tract-based spatial statistics (TBSS) approach was applied in FSL software (version 6.0; the Oxford Centre Functional Magnetic Imaging Software Library; Oxford, UK;^43^. Artefact correction for motion and Eddy-Current artefacts correction were executed for all subjects using the FDT software tool in FSL and data was projected to pre-determined brain masks to eliminate non-brain structures with an optimal fractional intensity threshold (FIT) of FIT = 0.3 for our sample using the brain extraction tool BET in FSL^44^. We inspected two major association fiber tracts (UF, cingulum) and one projection fiber tract (ATR) with predefined TBSS tract masks. For these tracts fractional anisotropy (FA) the most common parameter in DTI analyzes^45^ was estimated. We additionally calculated radial and axial diffusivity (RD and AD) for regions with significant FA clusters.

Finally, non-linear registration via standard Montreal Neurological Institute space (MNI-152;^46^) transformation was used for all three parameters to receive standardized data with a mean skeleton each parameter (threshold < 0.2) to avoid errors of wrong alignment.

### Statistics

For all analyses, we conducted randomized voxel-wise analyses with Threshold-Free-Cluster-Enhancement (TFCE) and family-wise-error (FWE) correction for multiple comparisons with a threshold of *p* < 0.05. We generated F-contrasts with 5000 permutations^47^.

We tested our hypotheses using the general linear model (GLM) approach in FSL^43^ with multiple regression analyses. We set up separate models for PNI scales (*Vulnerable, Grandiose* and *total*) whereby age, sex, and TIV served as nuisance regressors. Contrasts were tested with FA which was defined by a tract- mask of cingulum, ATR, and UF.

For additional explorative analyses we used the same approach to investigate FA of UF, cingulum, and ATR in dependence of the seven PNI subscales (CSE, DEV, EXP, ENTR, GRAND, HIDE, and SSSE) with sex, age, and TIV as nuisance variables.

Additionally, we conducted sex- specific analyses with the same methodological approach. We only included clusters with > 50 voxel in our results.

### Ethics approval

The study was approved by the local Ethics Committee of the school of medicine, Philipps-University Marburg, and conducted according to the current version of the Declaration of Helsinki (World Medical Association, 2013).

### Informed consent

All participants gave written informed consent before inclusion in the study and were financially compensated afterwards.

## Results

### Correlation of PNI total, grandiose, and vulnerable scale with FA

**Figure 2:**
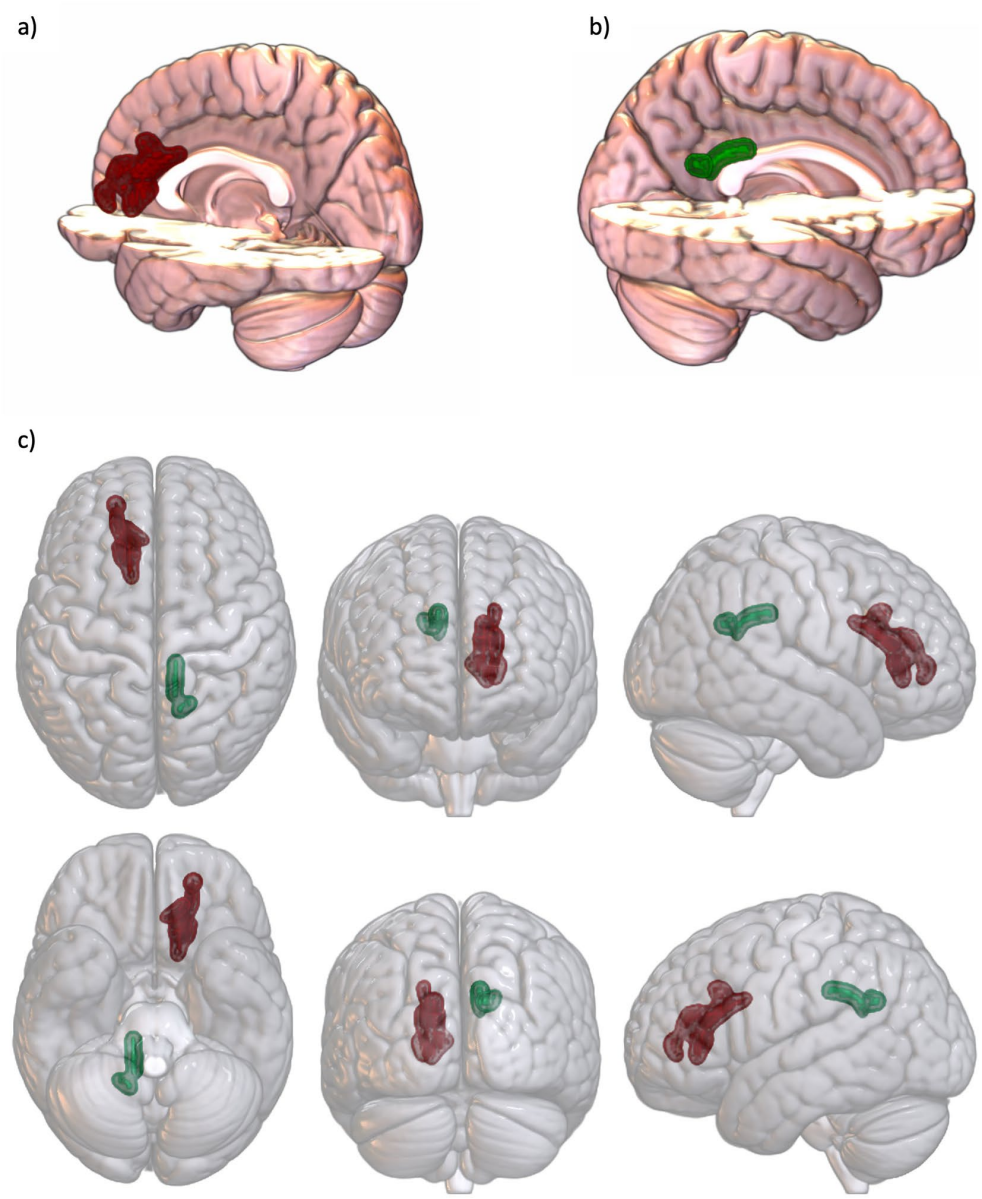
Association of PNI scales and FA (three-dimensional). Three-dimensional view of PNI Vulnerable and association with FA (all FWE peak level corrected); red: significant clusters left anterior cingulum, green: significant clusters right posterior cingulum; cluster edges are displayed enlarged; depiction made with MRICroGl (version 12.3.1, https://www.nitrc.org/projects/mricrogl); (**a**) PNI Vulnerable & right posterior cingulum cut-out; (**b**) PNI Vulnerable & left anterior cingulum cut-out; (**c**) PNI Vulnerable & left anterior and right posterior cingulum.

FA was positively correlated with PNI *total* in right posterior cingulum (*p* = 0.03 at FWE peak level) and PNI *Vulnerable* in left anterior cingulum (*p* = 0.02 at FWE peak level) and right posterior cingulum (*p* = 0.03 at FWE peak level). More detailed information about location and cluster size are illustrated in Table 2. Results are presented graphically in Fig. 1 and 2 No significant correlations were detected for PNI *Grandiosity*.

**Table 2:** Overview of coordinates and anatomical labels for multiple regression analyses of FA (*p* < 0.05 FWE peak level) with PNI Total and Vulnerable scale (k = number of voxels).

	Correlation	Coordinates	Anatomical	k
			Labels	
Vulnerable	Pos	− 0.0261	Left anterior cingulum	223
	Pos	15/− 53/26	Right posterior cingulum	14
Total	Pos	15/− 53/26	Right posterior cingulum	9

### Correlation of FA and PNI subscales

Additionally, exploratory analyses of subscales detected positive correlations of FA and DEV in two clusters in left anterior cingulum (*p* = 0.01; *p* = 0.03 at FWE peak level). Furthermore, another positive effect was found for FA and ENTR in left anterior cingulum (*p* = 0.004 at FWE peak level). Further details are presented in in supplemental material (Fig. 2 and 3).

### Sex-specific analyses

Correlations of PNI scales with sex and other demographic variables are given in Table 3. Comparison of psychometric data for female and male participants showed significant group level differences for PNI *Grandiosity* (male > female; t = -2.17, *p* = 0.03), PNI CSE (female > male; U = 9802.5, *p* = 0.01) and PNI EXP (male > female; U = 5593.5, *p* < 0.001). All correlations are presented in Table 4.

**Table 3:** Correlation of PNI main scales with demographic variables.

PNI scale	Age		Sex		Education		Occupation	
	r	*p*	R	*p*	r	*p*	r	*p*
**Total**	** − 0.14**	**0.02**	< 0.00	0.96	0.01	0.89	− 0.04	0.51
**Grandiosity**	** − 0.17**	**0.01**	**0.13**	**0.03**	0.03	0.60	− 0.01	0.91
**Vulnerable**	− 0.10	0.11	− 0.07	0.25	− 0.01	0.85	− 0.06	0.33
CSE	− 0.10	0.09	** − 0.17**	**0.01**	0.02	0.80	− 0.05	0.46
EXP	− 0.06	0.32	**0.29**	** < 0.001**	− 0.02	0.73	**0.12**	**0.04**
DEV	− 0.01	0.90	− 00.02	0.71	− 0.07	0.28	− 0.03	0.59
SSSE	** − 0.20**	** < 0.001**	− 0.04	0.52	0.02	0.78	− 0.08	0.17
GRAND	** − 0.13**	**0.04**	0.08	0.19	0.07	0.23	− 0.05	0.45
ENTR	** − 0.13**	**0.03**	− 0.06	0.36	< 0.00	0.99	− 0.03	0.62
HIDE	− 0.08	0.19	0.01	0.82	0.01	0.90	− 0.09	0.17

r: Pearson correlation coefficient, statistically significant Pearson correlation coefficients and *p*-values are in bold.

**Table 4:** Comparison of psychometric data for female and male participants.

Variable	M (SD)		Skewness		Kurtosis		statistics	
	Female	Male	Female	Male	Female	Male	T/U	*p*
PNI Total	2.57 (.76)	2.57 (.62)	0.16	0.11	− 0.55	− 0.19	t = 0.05	0.96
**Grandiosity**	**20.80 (0.70)**	**30.00 (0.76)**	** − 0.03**	**0.08**	** − 0.37**	**0.13**	**t = − 20.17**	**0.03**
Vulnerable	20.43 (0.74)	20.33 (0.64)	0.39	0.17	− 0.40	− 0.57	U = 8767	0.36
**CSE**	**20.62 (0.90)**	**20.32 (0.78)**	**0.35**	**0.39**	** − 0.49**	** − 0.41**	**U = 98,020.5**	**0.01**
**EXP**	**20.38 (0.77)**	**20.89 (0.91)**	**0.49**	**0.44**	** − 0.27**	** − 0.33**	**U = 55,930.5**	** < 0.001**
DEV	20.08 (0.82)	20.04 (0.77)	0.78	0.52	0.33	− 0.36	U = 83,500.5	0.81
SSSE	30.31 (0.88)	30.23 (0.97)	− 0.08	− 0.23	− 0.35	− 0. 46	U = 8463	0.67
GRAND	20.69 (0.97)	20.86 (0.96)	0.30	0.45	− 0.56	0.02	U = 74,444	0.21
ENTR	20.39 (0.90)	20.29 (0.72)	0.50	0.37	− 0.57	− 0.24	U = 84,410.5	0.70
HIDE	20.63 (0.95)	20.65 (0.83)	0.62	0.22	− 0.10	− 0.43	U = 78,260.5	0.53c

CSE: Contingent-Self-Esteem, EXP: Exploitative, DEV: Devaluing, SSSE: Self-sacrificing-self-enhancement, GRAND: Grandiose Fantasy, ENTR: Entitlement rage, HIDE: Hiding the Self , n = 267, 171 = female, 96 = male; Statistically significant group differences are in bold.

We found a significant sex-specific effect in FA of left ATR for PNI EXP (*p* = 0.01 at FWE peak level) with a negative association for females and positive for males (coordinates of maximum voxel intensities [99, 105, 77]; k = 51). The supplemental material Fig. 4 contains a figure with relevant brain regions and a scatter plot.

## Discussion

The present study was conducted to test the hypothesis that non-clinical narcissistic traits are associated with structural connectivity in fronto-thalamic and fronto-limbic pathways. These tracts are related to major networks involved in cognitive and emotional evaluation. Our DTI analyses provide an additional gain besides voxel- and surface-based analyses to develop a network-oriented theory for narcissistic traits. Moreover, this study is the first to investigate both grandiose and vulnerable aspects of narcissism in a large psychiatrically healthy sample.

One prominent feature of fronto-limbic networks is their relation to emotion regulation^48–50^ which is a core feature of narcissism^16^ and has been emphasized in previous imaging studies^24,25^. Our findings suggest a positive association of FA in dorso-limbic areas (left anterior and right posterior cingulum) and vulnerable narcissistic traits. These results can be interpreted as a demand of managing negative feelings like anger, rage, and shame which are associated with narcissistic vulnerability^16^. More precisely, anterior sections are assumed to refer to emotional appraisal (connections to amygdala and OFC;^51,52^) and could explain our significant correlations with sub-scales.

entitlement rage and devaluing Whereas posterior parts are supposed to be involved in spatial orientation and memory (relation to hippocampus and parietal cortex) which is demanded for extracting personally relevant stimuli from the environment^52^.

Our analyses failed to find correlations in FA in ventro-limbic networks (UF) which connect orbitofrontal with anterior temporal cortex and are essential for emotion regulation as well^53,54^. Though it should be noted that dorso-limbic networks that are also related to cognitive control, action–outcome learning, and episodic memory.

However, we cannot assume for certain that our results are inevitably to be interpreted as relating to emotion regulation or cognitive control given the fact that we focused on large neural networks with broad ranges of functions^49,52–55^. Furthermore, we only performed association analyses which cannot provide information about possible mediating or moderating variables. Nevertheless, our results are in support of previous findings of vulnerable narcissism/social subordination. Additionally, changes in fronto-limbic networks were already investigated in narcissistic phenotype related constructs and like social dominance^5–7,56^ neuroticism^35,57^ emotional reactivity^28^ emotional and behavioral inhibition^58^, and negative emotional-related traits^36^.

Unlike other studies that addressed narcissistic related personality traits^35,57^ or even personality disorders^31,32^ we detected positive associations rather than negative ones. There is one aspect, which we take into consideration for explaining this aspect: the relationship of narcissistic traits and structural connectivity may follow a non-linear path with positive correlations in low-level narcissism and negative correlations in high-level narcissism. This phenomenon was already observed in other studies concerning narcissism and other related traits^24,59,60^.

We hypothesized associations in cortico-striato-thalamo-cortical circuits (CSTC) which have distinct loops that are associated with different aspects of motor and cognitive functions^61^. Fronto-thalamic fibers (ATR) which are located in anterior limb of capsula interna (ALIC;^62^) and connect mediodorsal thalamus with PFC^63^ have been associated with general cognitive functions^64,65^ whereby fronto-striatal networks are rather associated with self-reward-related aspects like self-esteem^33,34^) or social subordination^8^.

Our primary analyses did not affirm associations of narcissistic traits and FA in ATR. But additional sex-specific analyses indicate a correlation with aspects of grandiose narcissism (exploitation) which is positive in female and negative in male individuals.

Exploitative behavior is one of the core features of antisocial personality disorder (AsPD;^12^) and negative associations with FA in ALIC have been detected before^66^. Sex related differences in narcissistic traits were already identified for brain volume and functional connectivity^31^ and behavioral aspects^67^. However, it must again be emphasized that we focused on neural networks with many functions in cognitive domains^64,65^. Notwithstanding, our results underpin findings of non-clinical narcissism^33^ and NaPD^22,24,30^.

There are some limitations by interpreting the results of our study. First of all, the PNI has a strong focus on vulnerable traits and grandiose aspects are highly reduced^16^. Moreover, the grandiose and vulnerable scales are highly correlated (r = 0.63) and the operationalizing of grandiose narcissism differs from other assessment tools like NPI^16,20^.

Nevertheless, it is an assessment tool which includes both grandiosity and vulnerability compared to NPI. Another aspect that has to be considered is the nature of our sample which only includes psychiatrically healthy individuals, although PNI scores are somewhat higher than in the original German validation sample^13^. Another point to discuss is the use of different methods comparability of our results to previous studies; we used the PNI whereby others conducted NPI on their sample which only includes grandiose aspects of narcissism^33^. Furthermore, we used TBSS to analyze DTI data, which allows us the use of predefined tract skeleton mask where subject’s data is projected while others used probabilistic tractography approach^33^.

In summary, our results make an important contribution to previously published studies as it is the first large analysis demonstrating differences in fronto-limbic and fronto-thalamic networks in healthy individuals and examines narcissism as a multidimensional construct. Further analyses are needed to investigate different facets of narcissism in detail and white matter integrity, mediating or moderating effects, as well as structural connectivity.

### Supplementary Information

Supplementary Information.

## Data Availability

Data are available upon reasonable request (from I.N.) and pending local and national regulations.
