# Supplementary material for: Structural connectivity of grandiose versus vulnerable narcissism as models of social dominance and subordination
Source: Sci Rep. 2023 Sep 26;13:16098. doi: 10.1038/s41598-023-41098-1 (PMC10522767; doi:10.1038/s41598-023-41098-1)

Supplement

Tables

Table 1.
Overview of coordinates and anatomical labels for multiple regression analyses of FA ($p < 0.05$ FWE peak level) with subscales PNI Devaluing and PNI Entitlement Rage; k = number of voxels

	correlation	*coordinates*	*anatomical labels*	*k*
Devaluing	pos.	-13 / 24 / 19	left anterior cingulum	413
	pos.	-10 / 30 / 10	left anterior cingulum forceps minor	120
Entitlement Rage	pos.	-10 / 28 / 14	left anterior cingulum	535

Figures

Figure 1.
Inter-correlation of PNI scales; Pearson correlation; significance level is marked by stars (*** = p <= 0.001, ** = p <= 0.01, * = p <= 0.05); depiction made with R Studio (version 2022.2.3.492, https://www.rstudio.com/)

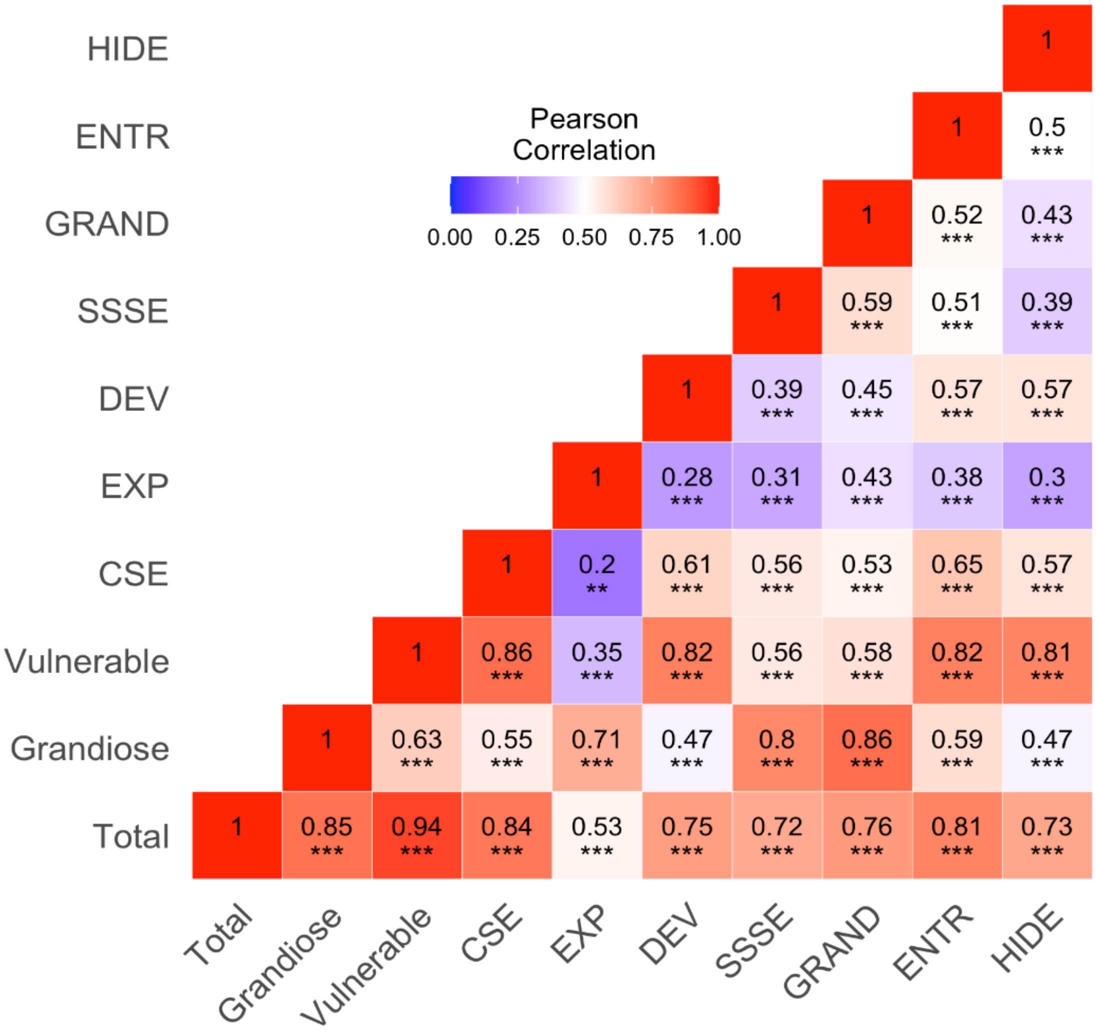

Figure 2.

PNI Devaluation and Entitlement Rage scale and association with FA (all FWE peak level corrected) and scatter plots (made with R Studio (version 2022.2.3.492, https://www.rstudio.com/); yellow: tract mask; red: significant clusters; cluster edges are displayed enlarged; depiction made with MRICroGl (version 12.3.1, https://www.nitrc.org/projects/mricrogl); a) PNI DEV & left anterior cingulum cluster I; b) PNI DEV & left anterior cingulum / forceps minor; c) PNI ENTR & left anterior cingulum

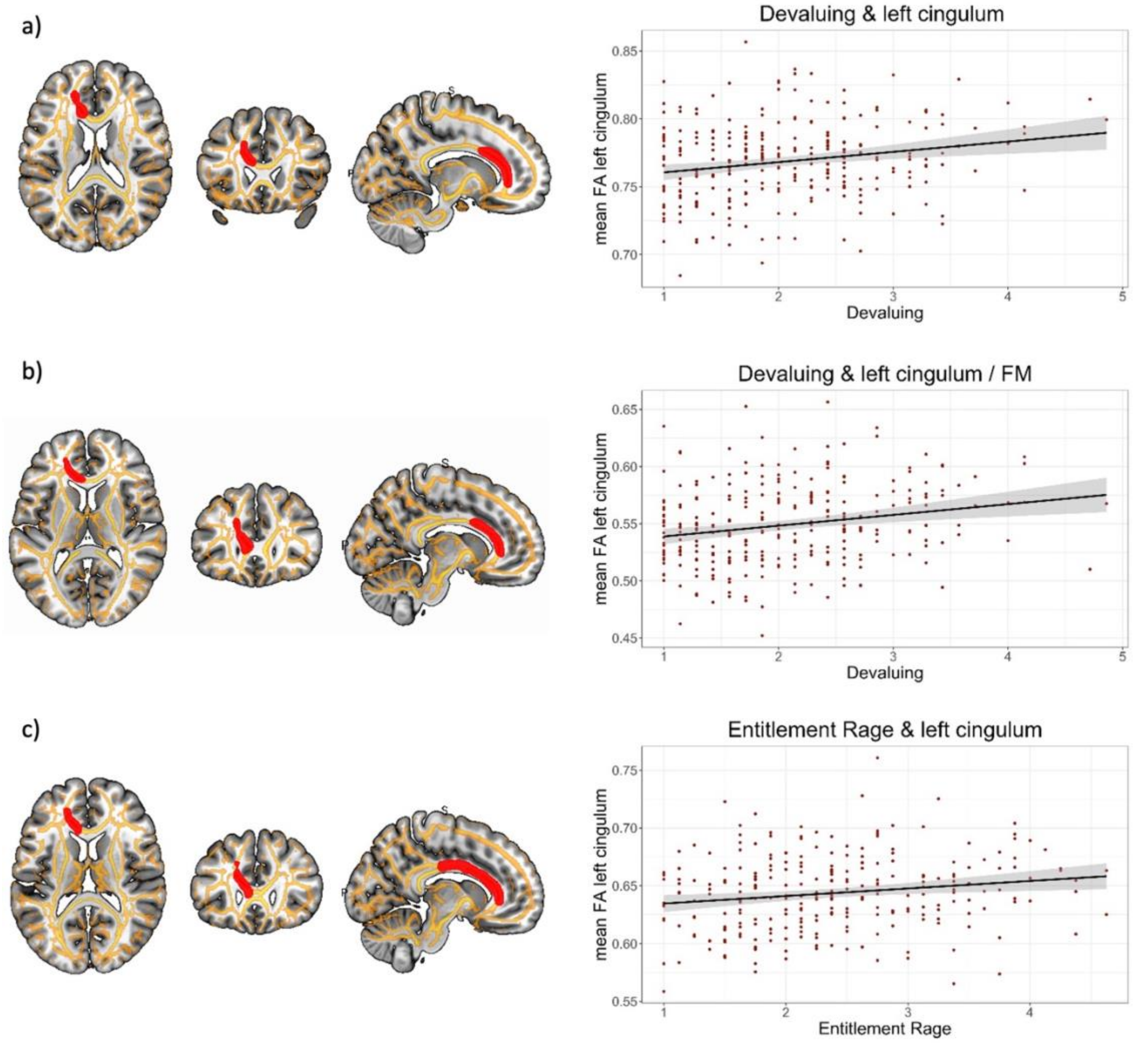

Figure 3.

Three-dimensional view of PNI Devaluation and Entitlement Rage scale and association with FA (all FWE peak level corrected); red: significant clusters; cluster edges are displayed enlarged; depiction made with MRICroGl (version 12.3.1, https://www.nitrc.org/projects/mricrogl); a) PNI DEV & left anterior cingulum cut-out; b) PNI ENTR & cut-out; c) PNI DEV & left anterior cingulum; d) PNI ENTR & left anterior cingulum

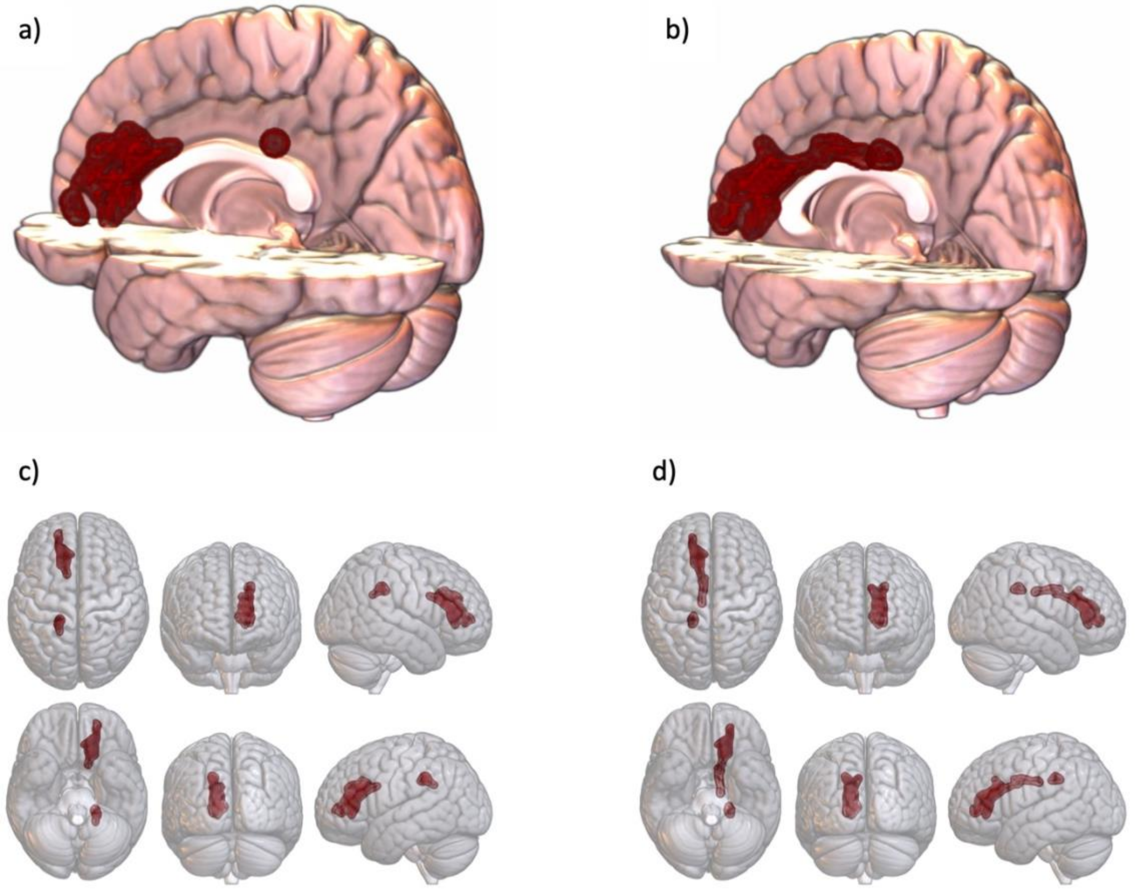

Figure 4.

Sex-specific effect of PNI Exploitative and association with FA in left ATR (all FWE peak level corrected) and scatter plot (made with R Studio (version 2022.2.3.492, https://www.rstudio.com/); yellow: tract mask; red: significant clusters; cluster edges are displayed enlarged; depiction made with MRICroGl (version 12.3.1, https://www.nitrc.org/projects/mricro

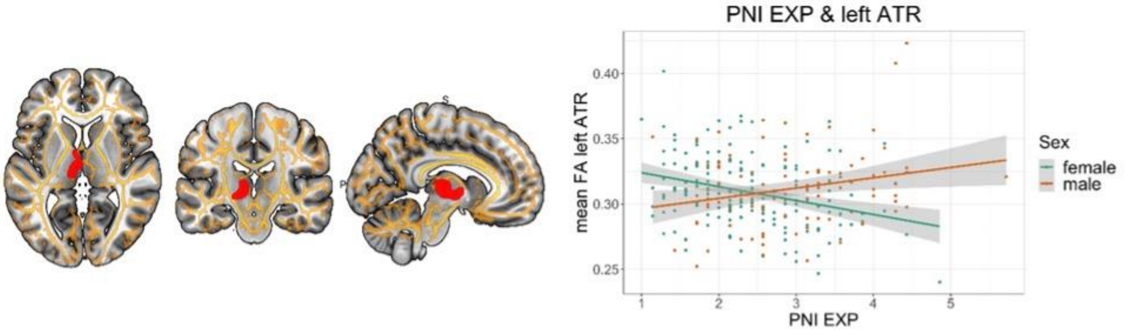

Supplement: Supplementary file 1 — Supplementary Information. [file 41598_2023_41098_MOESM1_ESM.pdf]
